# Amygdala responses to unpleasant pictures are influenced by task demands and positive affect trait

**DOI:** 10.3389/fnhum.2015.00107

**Published:** 2015-03-04

**Authors:** Tiago A. Sanchez, Izabela Mocaiber, Fatima S. Erthal, Mateus Joffily, Eliane Volchan, Mirtes G. Pereira, Draulio B. de Araujo, Leticia Oliveira

**Affiliations:** ^1^Laboratório de Neuroimagem Convencional e Avançada, Departamento de Radiologia, Faculdade de Medicina, Universidade Federal do Rio de JaneiroRio de Janeiro, Brazil; ^2^Polo Universitário de Rio das Ostras, Universidade Federal FluminenseRio das Ostras, Brazil; ^3^Instituto de Biofísica Carlos Chagas Filho, Universidade Federal do Rio de JaneiroRio de Janeiro, Brazil; ^4^Groupe d’Analyse et de Theorie Economique, Centre National de la Recherche ScientifiqueLyon, France; ^5^Instituto Biomédico, Universidade Federal FluminenseNiteroi, Brazil; ^6^Instituto do Cérebro/Hospital Universitário Onofre Lopes, Universidade Federal do Rio Grande do NorteNatal, Brazil

**Keywords:** amygdala, attention, emotion, emotion regulation, positive affect, fMRI, PANAS

## Abstract

The role of attention in emotional processing is still the subject of debate. Recent studies have found that high positive affect in approach motivation narrows attention. Furthermore, the positive affect trait has been suggested as an important component for determining human variability in threat reactivity. We employed functional magnetic resonance imaging to investigate whether different states of attention control would modulate amygdala responses to highly unpleasant pictures relative to neutral and whether this modulation would be influenced by the positive affect trait. Participants (*n* = 22, 12 male) were scanned while viewing neutral (people) or unpleasant pictures (mutilated bodies) flanked by two peripheral bars. They were instructed to (a) judge the picture content as unpleasant or neutral or (b) to judge the difference in orientation between the bars in an easy condition (0 or 90^∘^ orientation difference) or (c) in a hard condition (0 or 6^∘^ orientation difference). Whole brain analysis revealed a task main effect of brain areas related to the experimental manipulation of attentional control, including the amygdala, dorsolateral prefrontal cortex, and posterior parietal cortex. Region of interest analysis showed an inverse correlation (*r* = -0.51, *p* < 0.01) between left amygdala activation and positive affect level when participants viewed unpleasant stimuli and judged bar orientation in the easy condition. This result suggests that subjects with high positive affect exhibit lower amygdala reactivity to distracting unpleasant pictures. In conclusion, the current study suggests that positive affect modulates attention effect on unpleasant pictures, therefore attenuating emotional responses.

## INTRODUCTION

Humans exhibit involuntary co-occurring brain and peripheral responses to emotional stimuli, such as faces with fearful expressions or unpleasant pictures (Schneider et al., 1995; Cuthbert et al., 2000; Hagemann et al., 2003). Emotion is believed to take place automatically in the amygdala, independent of top–down factors such as attention and awareness (Vuilleumier et al., 2001; Anderson et al., 2003; Muller et al., 2008). However, the role of attention in emotional processing is still the subject of debate. Growing evidence indicates that affective processing is modulated by several factors, including attention and cognitive regulation (Erthal et al., 2005; Ochsner and Gross, 2005; Pessoa et al., 2005; Mocaiber et al., 2010, 2011). Emotional modulation by attention may be achieved through mechanisms associated with attention selection and by manipulating the strength of object representations (Mitchell et al., 2007). For example, manipulating the focus of spatial attention has been shown to eliminate differential signals evoked by fearful faces in both functional magnetic resonance imaging (fMRI) and event-related potential (ERP) studies (Pessoa et al., 2002, 2005; Eimer et al., 2003).

Currently, the reasons for this discrepancy are unclear, suggesting that other variables may contribute to this effect (see Oliveira et al., 2013). One possibility is that individual differences are important predictors of sensitivity to emotional stimuli. For instance, anxious individuals exhibit greater sensitivity to threat-related stimuli, and the extent to which the amygdala responds to threat-related distractors depends on individual anxiety levels (Bishop et al., 2004). Whereas low-anxiety individuals only show increased amygdala responses to attended fearful faces, high-anxiety individual’s show increased amygdala responses to both attended and unattended threat-related stimuli. These findings suggest that the threat value of a stimulus varies as a function of a participant’s anxiety level, although attention is important even for highly anxious individuals (Fox et al., 2005; Bishop et al., 2007).

Similarly, it is possible that individual differences in the positive affect trait could modulate the emotional reactivity to threat-related stimuli. In general, positive affect reflects the extent to which a person feels enthusiastic, active, and alert. Therefore, high positive affect is a state of high energy, full concentration, and pleasurable engagement, whereas low positive affect state is characterized by sadness and lethargy (Watson et al., 1988). Positive affect trait is likely an important component for determining human variability in threat perception and an enhanced ability to disengage attention from unpleasant stimuli (Segerstrom, 2001; Isaacowitz, 2005). Previous study from our group showed that participants with high trait of positive affect were more prone to be engaged by safety cues. Specifically, their psychophysiological reactions to mutilated body pictures were attenuated in an experimental context in which these pictures were presented as makeup tricks used to mimic wounds in movie productions (Oliveira et al., 2009). In fact, Individuals with high levels of positive affect experience more persistent positive mood and they are more actively engaged in the world, showing a predominant approach disposition and high reward sensitivity (Whittle et al., 2006).

In the current study, we investigated whether individual differences in the positive affect trait modulate attention resources to process unpleasant stimuli. Our hypothesis is that the high positive trait facilitates disengagement from unpleasant stimuli. More specifically, we aimed to test whether positive affect diminishes the interference produced by unpleasant stimuli (mutilated pictures) presented as a distractor during the performance of an attentional task. Thus, considering that the amygdala response is a marker of the impact of an emotional stimulus and that it is modulated by attention, we employed fMRI to study the amygdala’s responses when subjects viewed highly unpleasant distractors during an attentional task.

## MATERIALS AND METHODS

### PARTICIPANTS

Twenty-two healthy right-handed volunteers between 19 and 37 years of age (12 male; mean age = 26.32, SD = 4.52 years) took part in this study. The volunteers were selected among students from the University of São Paulo, and had normal or corrected-to-normal vision. They reported no psychiatric or neurologic problems and were not under the influence of medications with nervous system actions. The subjects were naive to the purpose of the experiment. The local ethical committee approved this study, and written informed consent was obtained from all the volunteers.

### POSITIVE AFFECT TRAIT

The Positive and Negative Affect Schedule PANAS is a 20-item scale, consisting of 10 adjectives that describe positive and negative moods (Watson et al., 1988). In the current study, the PANAS was used to evaluate individuals’ mean positive affect trait. The participants were asked to fill out this scale before the fMRI session, when they rated the degree to which they felt each emotion on a 1–5 scale (1 = very slightly or not at all, 5 = extremely).

### EXPERIMENTAL PROCEDURE

The subjects were scanned while viewing neutral (people) or unpleasant pictures (mutilated bodies). Each of these images was centrally presented with two bars on its periphery. They had to perform three discrimination tasks: (a) judge the picture content by its valence as unpleasant or neutral, or (b) judge the difference in orientation of the bars in an easy condition (0 or 90^∘^ orientation difference) or (c) in a hard (0 or 6^∘^ orientation difference) condition. The participants performed the task by pressing one of two buttons to report whether the picture presented was neutral or negative during the emotional judgment task or whether the bars had the same or different orientation during the bar orientation tasks. In task (a), the subjects had their attentional resources engaged centrally to evaluate the valence of the picture, while in task (b) and (c) the subjects had their attentional resources engaged to judge the peripheral bars (away from the emotional stimuli). Furthermore, task (c) demanded an extra attention load due to its increased difficulty.

**FIGURE 1:**
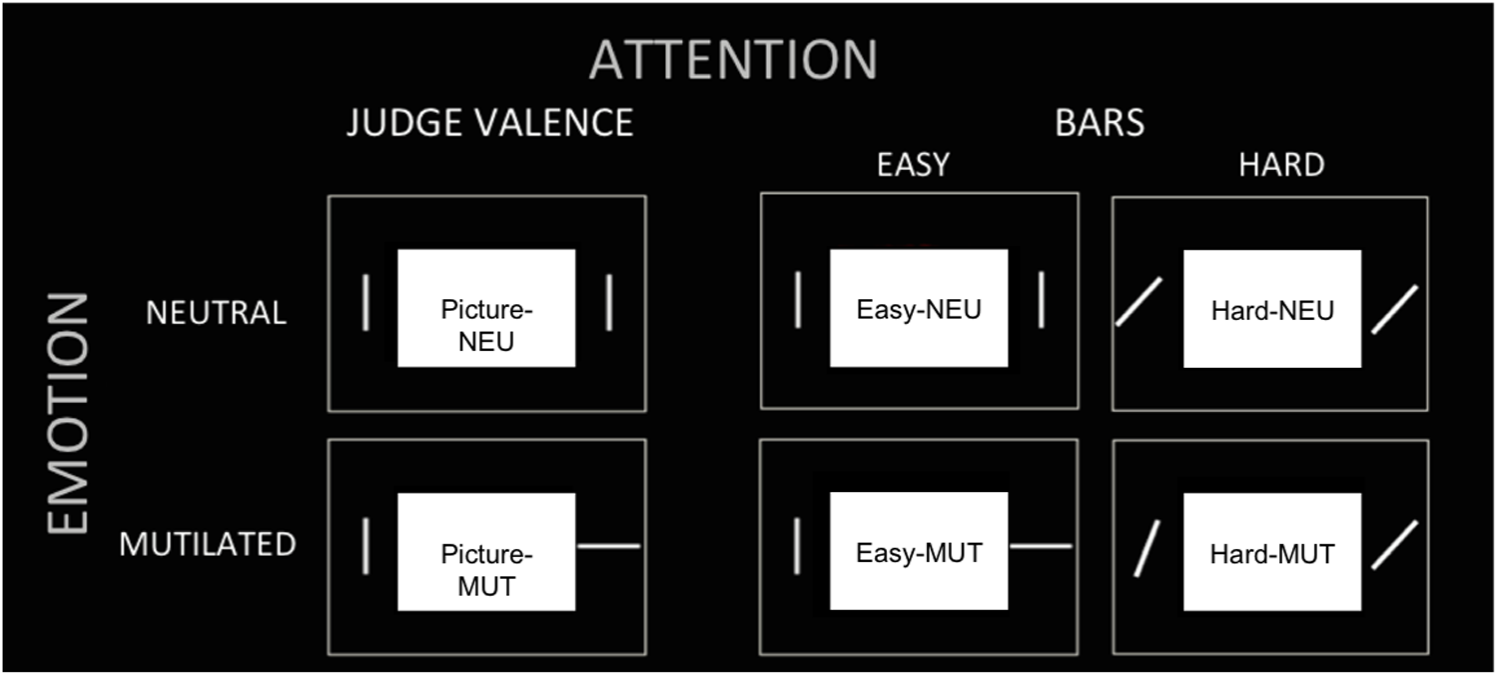
**Experimental conditions.** The tasks (three columns) and picture valence (two rows) are presented to illustrate the six experimental conditions: (1) “Picture NEU,” (2) “Picture MUT,” (3) “Easy NEU,” (4) “Easy MUT,” (5) “Hard NEU,” (6) “Hard MUT.”

In summary, there were three tasks (judgment of “Pictures,” judgment of “Easy Bars” and judgment of “Hard Bars”). In each task, a neutral or mutilated body picture was presented. Then, there were six experimental conditions, which were designated as follows: (1) “Picture NEU,” the task of judging neutral valence pictures; (2) “Picture MUT,” the task of judging mutilated body pictures; (3) “Easy NEU,” the task of judging easy orientation bars when the picture was neutral; (4) “Easy MUT,” the task of judging easy orientation bars when the picture was a mutilated body picture; (5) “Hard NEU,” the task of judging hard orientation bars when the picture was neutral; and (6) “Hard MUT,” the task of judging hard orientation bars when the picture was a mutilated body picture (see **Figure 1**).

The protocol followed a mixed blocked/event-related design (Donaldson, 2004; Amaro and Barker, 2006). It consisted of two runs, each with 12 randomly distributed blocks of the three tasks (“Picture,” “Easy Bar,” and “Hard Bar”), alternating with periods of rest (central fixation cross). During the task periods, the subjects carried out nine trials with either unpleasant or neutral pictures presented (**Figure 1**). Each trial lasted 3 s and was initiated with a fixation cross, shown for 500 ms, followed by 200 ms of the picture/bars image and a gray scale checkerboard that remained until the volunteer responded. The subjects were instructed to respond as quickly and accurately as possible. Each run included an equal number of neutral and emotional trials.

The presentation of each picture across the different task conditions was randomized between subjects. Valence and arousal were balanced between different blocks. Therefore, the pictures presented to some participants in the picture judgment task were presented to the others in the bar orientation tasks.

### STIMULI

The stimulus protocols were generated on a PC laptop running the Presentation®; software (Version 0.60, Neurobehavioral Systems, http://www.neurobs.com/) and displayed using a projector and screen with a mirror system fixed on the head coil. The responses were collected with an MRI-compatible button system controlled by the right hand of the participant and registered by the Presentation®; software. The participants performed a training session prior to the experiment to ensure that they understood the experimental procedure.

Two classes of images (72 neutral and 72 unpleasant) were employed. The neutral pictures consisted of photographs of people “in normal life” and the unpleasant images consisted of photographs of mutilated bodies. Most of the pictures were selected from the International Affective Picture System (IAPS; Lang et al., 2005). A set of additional images was obtained from the World Wide Web or photographed by the authors because the number of appropriate images available in the IAPS set was not sufficient. These were matched to the IAPS unpleasant and neutral stimuli in terms of color spectrum and complexity (e.g., number of faces, number of body parts, etc.). Following the protocol developed by Lang et al. (2005), all of the images were assessed on a 1–9 scale in terms of valence (from negative to positive) and arousal (from low to high) by a separate group of participants (*n* = 20) with ages similar to the subjects of the current study (22.3, SD = 1.8). The unpleasant and neutral images differed significantly from each other in IAPS normative valence (*M* = 2.08 and 5.21, respectively, *t* = -58.02, *p* < 0.001) and arousal (*M* = 6.6 and 3.4, respectively, *t* = 34.43, *p* < 0.001) ratings. Unpleasant pictures with high arousal were selected in order to maximize the interference effect and brain activation to these pictures. Each picture was repeated once per block of the same experimental condition.

### IMAGE ACQUISITION

The fMRI data were collected using a 1.5 T MRI scanner (Magnetom Vision; Siemens Medical Systems, Erlangen, Germany). The functional images were acquired using a gradient-echo planar imaging sequence (TR = 3000 ms; TE = 60 ms; FOV = 240; flip angle = 90^∘^; 64 × 64 matrix). Whole brain coverage was obtained with 25 axial slices (thickness = 4 mm; in-plane resolution = 3.75 mm × 3.75 mm). High-resolution structural T1-weighted images (TR/TE = 9.7/4.0 ms; flip angle = 12^∘^; 160 slices; thickness = 1 mm; 256 × 256 matrix; FOV = 256 mm) were obtained during the same session. The presentation of a stimulus was synchronized with the acquisition of an image using a triggering circuitry. The subjects’ head movements were restrained with foam padding.

### DATA ANALYSIS

#### Behavioral data

The latency of the correct responses (reaction time) was analyzed using Statistica^TM^ (7). Mean reaction times were determined for 19 of the 22 subjects; behavioral data from three subjects were not recorded due to technical problems. The reaction time analysis was performed with a two-way ANOVA with task (“Picture,” “Easy Bar,” and “Hard Bar”) and valence (neutral and mutilated) as within factors. Statistically significant effects identified through ANOVA were further evaluated *post hoc* using the Newmann–Keuls method for pairwise comparisons.

#### fMRI whole-brain analysis

The fMRI analysis was performed in BrainVoyager^TM^QX 2.2 (Brain Innovation, Maastricht, The Netherlands) using a general linear model (GLM). The dataset was corrected for motion and slice timing, and it was spatially filtered (8 mm FWHM) and temporally filtered (high pass filter at 0.01 Hz). Individual functional maps were normalized into the Talairach anatomical atlas (Talairach and Tournoux, 1988). After pre-processing, first-level analysis was performed on each subject using the GLM with a boxcar waveform convolved with a canonical hemodynamic response function. Six regressors of interest were created that correspond to the experimental conditions: Picture NEU, Picture MUT, Easy NEU, Easy MUT, Hard NEU, and Hard MUT.

After transformation into Talairach anatomical atlas, random effects group analysis was calculated using an ANOVA in a whole-brain voxelwise approach. The statistical threshold was set to *p* < 0.05, with FDR corrected for multiple comparisons [q(FDR) < 0.05]. Only clusters of at least 50 mm^3^ were considered for further interpretation.

The fMRI whole-brain analysis was performed with a two-way ANOVA with two factors: task (“Picture,” “Easy Bar,” and “Hard Bar”) and valence (neutral and mutilated). ANOVA results in interaction or main effect of task. The null hypothesis for interaction would reveal that the differences between the attention tasks are consistent for neutral and unpleased pictures. Main effect of task represents the differences among tasks performed by participants: Picture task, Easy bar task, and Hard bar task. Statistically significant effects identified through ANOVA were further evaluated *post hoc* using the Newmann–Keuls method for pairwise comparisons.

#### Region of interest (ROI) analysis

Our *a priori* hypothesis involved the effects of attention manipulation on the activation of the amygdala in response to unpleasant pictures. Therefore, this structure was chosen as the main region of interest (ROI). ROI analysis using planned comparisons in ROIs of bilateral amygdala was done to test differential activation between unpleasant and neutral pictures in each attention condition. Differential activation could reveal a valence effect during the picture judgment task (comparing Pic-MUT vs. Pic-NEU), and the bar judgment in both the easy task (comparing Easy-MUT vs. Easy-NEU) and the hard task (comparing Hard-MUT vs. Hard-NEU).

The amygdala ROIs were defined including all the voxels presented on a cluster activation in prior ANOVA main effect of task [q(FDR) < 0.05] and extracting only the anatomical coordinates corresponding to Amygdala in Talairach atlas (Poldrack, 2006). Beta weights averaged across all voxels within each ROI were extracted for each experimental condition. Furthermore, pairwise comparison between mean beta weights for each condition were done using Student’s *t*-test and *p* < 0.05 was considered significant. The ROI anatomical coordinates are presented on **Table 1** and the cluster size for each ROI is: Left Amygdala = 908 voxels; Right Amygdala = 866 voxels.

**Table 1:** Areas modulated by attention during the tasks (ANOVA main effect of task) considering a threshold of 50 continuous voxels and qFDR < 0.05.

Region	Hemisphere	BA	*x*	*Y*	*z*	*S*_x_	*S*_y_	*S*_z_
Precentral gyrus	Left	4,6	-33	-15	52	5	4	4
	Right	4,6	42	-6	49	12	5	7
Postcentral gyrus	Left	2,3,40	-48	-25	47	7	5	6
	Right	1,2,3,40	50	-27	41	7	3	5
Superior frontal gyrus	Left	6,9	-9	15	47	13	12	9
	Right	6,8,9,10	26	31	43	11	19	14
Cingulate gyrus	Left	32	-3	17	40	1	2	1
	Right	32	7	17	37	4	13	4
Middle frontal gyrus	Left	6,8,9,10,11,47	-38	31	22	6	17	24
	Right	6,8,9,10,46	36	22	42	10	20	12
Inferior parietal lobule	Left	40	-49	-38	42	8	7	4
	Right	40	45	-40	44	6	8	5
Precuneus	Left	7,19	-13	-68	41	8	7	5
	Right	7,19	14	-64	44	7	8	4
Posterior cingulate	Left	29,30	-7	-49	10	4	3	3
	Right	29,30	6	-49	12	3	3	4
Inferior frontal gyrus	Left	13,45,46,47	-44	23	-5	10	6	9
	Right	9,13,47	41	8	4	12	3	23
Claustrum	Left	-	-29	17	2	1	3	2
	Right	-	29	15	5	2	3	4
Middle occipital gyrus	Left	19	-42	-75	4	11	7	8
	Right	19,37	49	-69	6	3	2	2
Amygdala	Left	-	-23	-5	-15	3	2	3
	Right	-	24	-5	-15	3	2	3
Middle temporal gyrus	Left	19,21,22,39	-54	-35	-3	6	28	16
	Right	19,21,37,39	48	-41	1	4	28	15
Hippocampus	Left	-	-29	-16	-15	2	4	3
	Right	-	30	-16	-15	2	4	3
Fusiform gyrus	Left	20,36,37	-40	-33	-18	7	16	4
	Right	18,20,37	35	-37	-17	6	20	4
Insula	Left	13	-33	20	2	2	2	3
	Right	13	36	14	5	3	6	4

The Talairach coordinates correspond to the center of the cluster of activity with its respective SD.

We also evaluated the covariance (ANCOVA) in BrainVoyager^TM^ between the fMRI signals and the individual trait levels measured by PANAS. The amygdala fMRI responses were obtained using the calculated differences between beta fMRI estimates, using the GLM that represents emotional modulation between the following experimental conditions: (1) “Easy MUT” > “Easy NEU”, (2) “Hard MUT” > “Hard NEU”, and (3) Pic-MUT > Pic-NEU.

## RESULTS

### BEHAVIORAL RESULTS

The mean reaction times of the correct responses and accuracy are shown in **Table 2**. An ANOVA revealed a significant main effect of task (*p* < 0.02) but no interaction or main effect of valence. *Post hoc* *t*-tests showed that the overall performance in the Hard Bar task was significantly slower in comparison to both the Picture (*p* < 0.005) and the Easy Bar tasks (*p* < 0.01). Concerning the accuracy data, an ANOVA revealed a significant main effect of task (*p* < 0.001) and main effect of valence (*p* < 0.01) but no significant interaction between these factors. *Post hoc* *t*-tests showed no significant difference between Picture and Easy Bar tasks. However, Hard bars task was significantly different from the two others conditions (*p* < 0.001, for both comparisons).

**Table 2:** Mean reaction times (in milliseconds) and SD for each task per valence condition.

Attention-related task	Valence-related picture	Reaction times (ms)	SD	Accuracy (%)	SD
Picture judgment	Neutral	919.1	109.1	68.0	7.4
	Mutilated	922.0	113.3	74.7	4.9
Bar orientation task EASY	Neutral	932.9	106.6	74.1	7.1
	Mutilated	960.6	116.5	73.4	6.2
Bar orientation task HARD	Neutral	1019.3	191.1	52.8	9.1
	Mutilated	1029.1	213.0	56.7	11.5

There was no significant correlation between individual Positive Affect scores and reaction times when participants viewed unpleasant stimuli relative to neutral during the Easy Bar task (*r* = -0.26, *p* < 0.28), Hard Bar task (*r* = -0.17, *p* < 0.49) or during picture judgment task (*r* = -0.06, *p* < 0.81).

### fMRI WHOLE-BRAIN ANALYSIS

**FIGURE 2:**
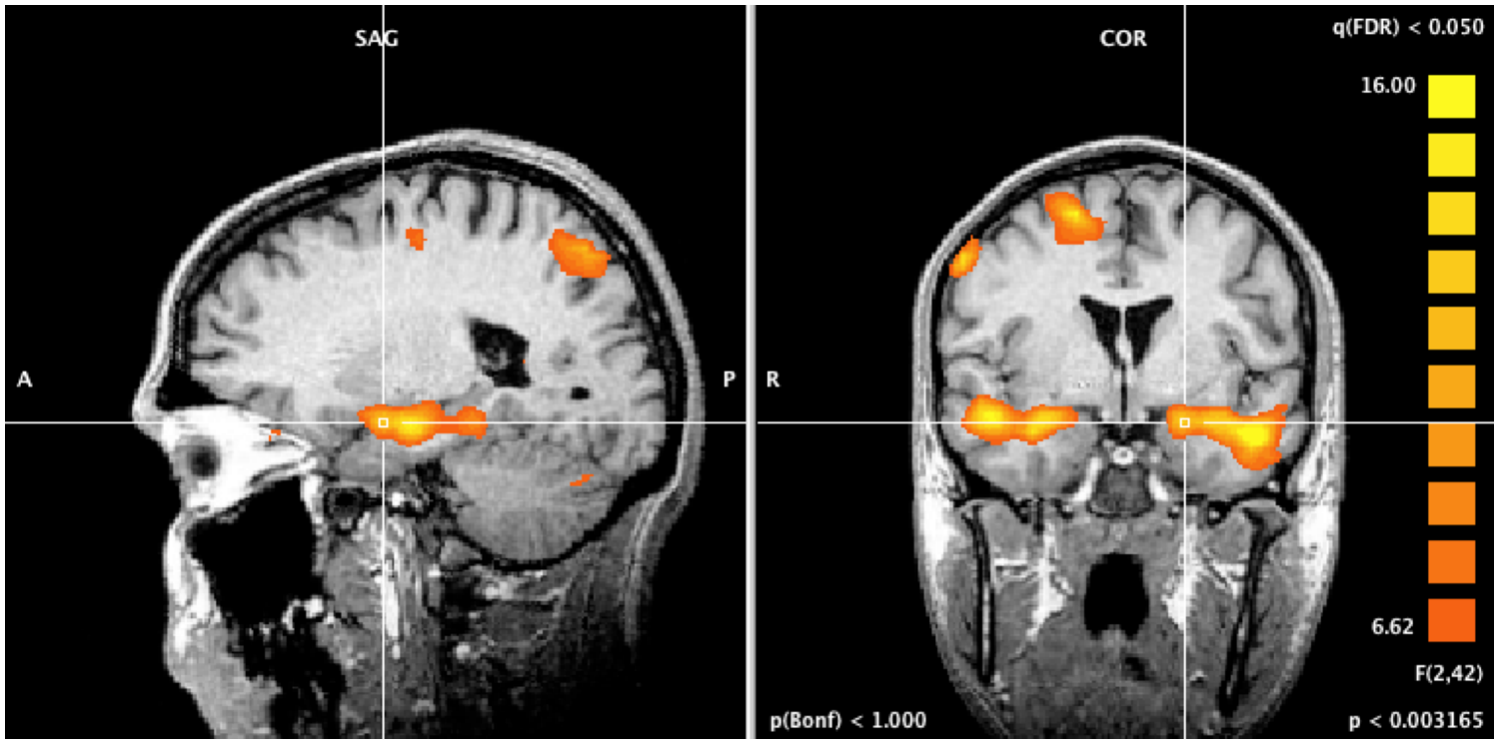
**Whole brain ANOVA, task main effect.** The crossed lines show the amygdala.

**FIGURE 3:**
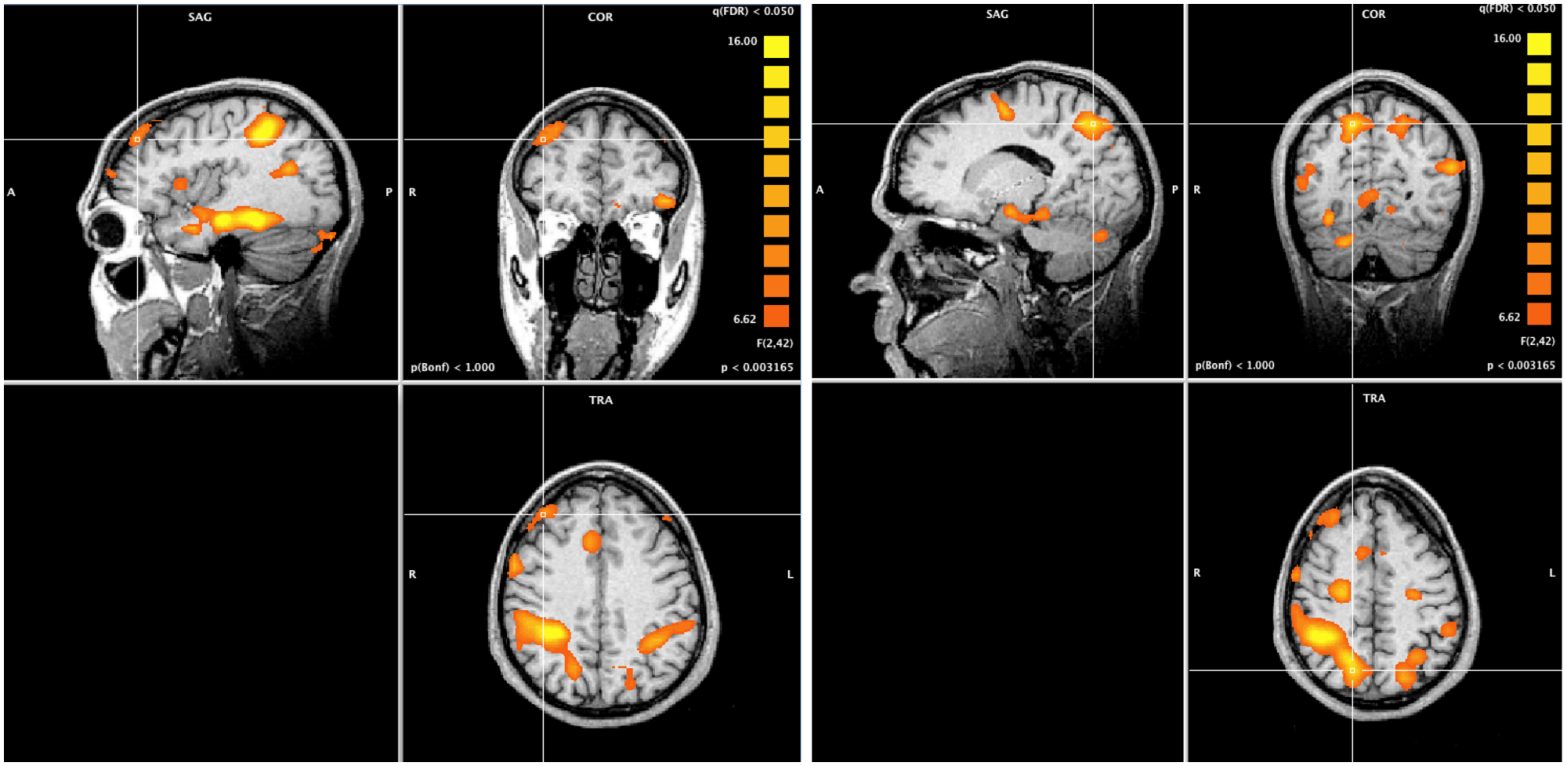
**Whole brain ANOVA, task main effect (qFDR < 0.05).** The crossed lines show the dorsolateral prefrontal cortex (left) and the posterior parietal cortex (right).

A whole-brain ANOVA analysis showed a main effect of task [q(FDR) < 0.05] for areas including the amygdala (**Figure 2**), the dorsolateral prefrontal cortex and the posterior parietal cortex (**Figure 3**). Moreover, a number of different clusters were also present in the main effect analysis (**Table 1**).

### ROI ANALYSIS

**FIGURE 4:**
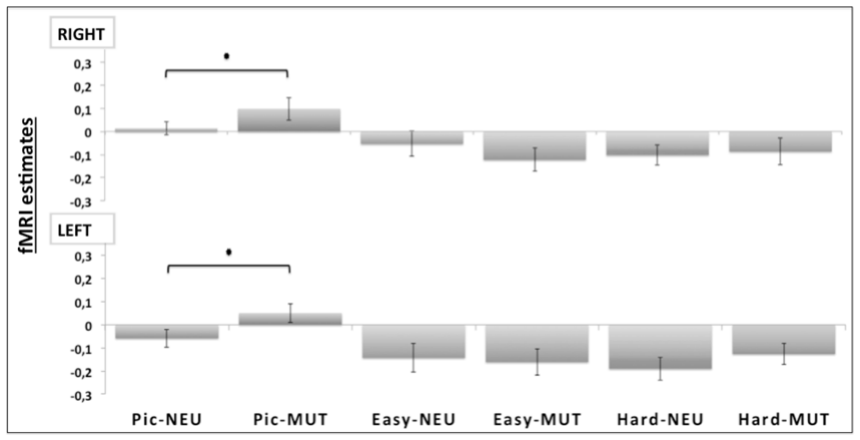
**Region of interest (ROI) analyses for the bilateral amygdala (RIGHT and LEFT).** Significant differences were observed bilaterally between the unpleasant pictures and the neutral conditions only for the picture judgment task (Pic-MUT vs. Pic-NEU; *p* < 0.05).

Region of interest analysis compared the bilateral amygdala beta values from the unpleasant and neutral pictures. Planned comparisons showed a valence effect only for the picture judgment task (Pic-MUT vs. Pic-NEU; *p* < 0.05; **Figure 4**). Such an effect was not observed when the participants judged the orientation of the bars, in both the easy (Easy-MUT vs. Easy-NEU; *p* < 0.19) and the hard conditions (Hard-MUT vs. Hard-NEU; *p* < 0.77).

### POSITIVE AFFECT TRAIT CORRELATIONS ON ROI ANALYSIS

**FIGURE 5:**
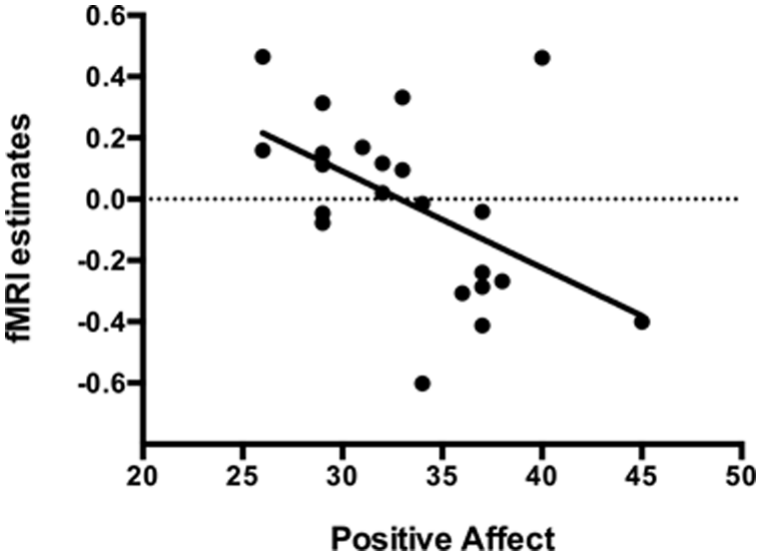
**Inverse correlation (*r* = -0.51, *p* < 0.01) between the individual left amygdala response to unpleasant stimuli with individual positive affect trait in the Easy bar orientation task**.

The mean positive affect trait was 33.32 (SD = 4.75), varying from 26 to 45. During the Easy Bar task a significant inverse correlation (*r* = -0.51, *p* < 0.01) was observed between the individual left amygdala response to unpleasant stimuli (Easy-MUT vs. Easy-NEU) and the individual PANAS scores (**Figure 5**). Subjects that scored higher in the positive affect trait exhibited lower amygdala reactivity to unpleasant pictures relative to neutral, and subjects that scored lower in the positive affect trait exhibited higher amygdala reactivity to unpleasant pictures relative to neutral. There was no significant correlation between the right amygdala response and the PANAS scores during the Easy Bar task (*r* = -0.11, *p* < 0.63). Furthermore, there was no correlation between positive affect and either the left or right amygdala during the Hard and Picture judgment task.

## DISCUSSION

In the current study, we showed that differences in the positive affect trait modulate the impact of attention on brain processing of unpleasant pictures relative to neutral. In the Easy Bar orientation task we found a significant inverse correlation (*r* = -0.51, *p* < 0.01) between left amygdala activation and positive affect (see **Figure 4**). Participants with a higher positive affect trait exhibited lower amygdala reactivity to unattended unpleasant pictures whereas participants with lower scores showed higher amygdala reactivity. These results suggest that positive affect facilitates the disengagement of attention from highly unpleasant pictures, increasing attentional resources to perform the task at hand.

The task main effect and ROI analysis revealed a reduced response of the amygdala to unattended emotional stimuli during non-emotional tasks (**Figure 4**), which may reflect a cognitive modulation of the amygdala activation (Pessoa et al., 2005). In fact, the task main effect revealed the activation of a selective visuospatial attention network, particularly involving the posterior parietal cortex and dorsolateral prefrontal cortex (Desimone and Duncan, 1995; **Figure 3**). Several studies have shown that the dorsolateral prefrontal cortex and posterior parietal cortex are implicated in selective visuospatial attention (Liu et al., 2004; Nobre et al., 2004) and in emotion modulation by attention and cognition (Pessoa et al., 2005; Blair et al., 2007; Mitchell et al., 2007). Furthermore, the left dorsolateral prefrontal cortex is widely implicated in the executive control of attention (MacDonald et al., 2000), in manipulating representations of task-relevant stimuli at the expense of higher conflict among distracters and stimuli (Botvinick et al., 2004), and in the presence of threatening distracters (Bishop et al., 2004).

Although some studies have proposed that emotion automatically evokes amygdala responses (Vuilleumier et al., 2001; Anderson et al., 2003; Muller et al., 2008), growing evidence demonstrates that affective processing is modulated by several factors, including attention and cognitive regulation (Erthal et al., 2005; Ochsner and Gross, 2005; Pessoa et al., 2005; Mocaiber et al., 2010, 2011).

It has also been suggested that the interaction between attentional control and emotional processing depends on a number of additional variables, such as the relevance of distracting emotional stimuli, task difficulty, and individual differences, such as anxiety and positive affect levels (Oliveira et al., 2013). Moreover, previous studies revealed that the amygdala response to threat varies as a function of individual focused attention and anxiety levels. Highly anxious individuals have more difficulty disengaging from threat stimuli (Fox et al., 2005; Bishop et al., 2007). Another study suggested increased attentional dwelling time on emotional facial stimuli, relative to neutral faces, for participants with a heightened anxiety trait (Fox, 2002).

Our results suggest that individual differences in positive affect trait influence attention and adjust amygdalar responses to threat-related stimuli. Positive affect is an important component of human reactivity to threat (Oliveira et al., 2009). For instance, participants with the high positive affect trait showed attenuated autonomic reactions to threat-related mutilation pictures in a context in which the pictures were presented as fictitious, suggesting that positive affect facilitated engagement in safety context interpretation, therefore diminishing the emotional impact of those pictures (Oliveira et al., 2009).

It is notable that the association between positive affective trait and emotional reactivity to unpleasant pictures was lateralized to the left amygdala activation, only. In fact, the Valence-Specific Hypothesis (VSH) suggested that the left cerebral hemisphere is specialized for processing positive emotions (Ahern and Schwartz, 1979; Adolphs et al., 2001). Furthermore, high left frontal activity is associated with positive-related traits including positive affect (Tomarken et al., 1992). One possibility is that the positive affective trait has more influence to modulate the left-brain activations, adjusting the attention influence just to the left amygdala reactivity.

In general terms, experiences of positive affect prompt individuals to engage with their environment and activities (Fredrickson, 2001; Carver, 2003), which could be linked to increases in brain dopamine levels (Ashby et al., 1999). Furthermore, considering that high positive affect involves high concentration, pleasure and alertness (Watson et al., 1988), individuals in this state would have increased attentional control and engagement with the task, thus reducing neural resources available for emotional processing. Isaacowitz (2005) used eye tracking to investigate attentional preferences and showed that optimistic people, compared with pessimists, presented selective inattention to unpleasant skin cancer images. In the current study, individuals with the high positive affect trait seem to present increased attention in the bar-orienting tasks, disengaging from unattended aversive pictures. It is important to highlight that, considering the experimental design of the present study, it is not possible to disentangle unpleasant pictures disengagement from facilitated attentional processing to perform the bar-orienting task. In fact, both processes can be responsible for the results obtained.

Recent evidence of the positive affect interaction with attention comes from studies that use global–local visual processing paradigms to assess biases in attentional focus. Positive affect, particularly in individuals low in approach motivation, can suggest a comfortable, stable environment and allows for a broadening of attention and cognition, which may serve adaptive functions. However, broadening does not occur when positive affect individuals are high in approach motivation (Gable and Harmon-Jones, 2008; Harmon-Jones and Gable, 2009). Such positive affect often encourages specific action tendencies, such as tenacious goal pursuit, and an associated reduction in attentional breadth. This reduced attentional breadth may prove adaptive, as it assists in obtaining goals.

It is interesting to note that the emotional modulation found in the current study was dependent on the attentional resources available for the emotional distracters. The correlation between positive affect and amygdala activity was found only in the easy condition, when attentional resources were still available, but not in the hard orientation condition. Bishop et al. (2007) also found a positive correlation between the state of anxiety and amygdala reactivity to threat-related distractors under low- but not high-attentional load. The results from Bishop et al. (2007) already suggested that some attentional resources are required to reveal the influence of anxiety on the amygdalar reactivity to threat.

Behavioral analysis did not detect emotional interference in task performance. It is possible that the interference produced by aversive pictures leads to opposite effects on behavior. In fact, it has been suggested that pictures of mutilated people induce freezing reactions in humans (Azevedo et al., 2005; Volchan et al., 2011), with a significant reaction time increase (Pereira et al., 2006, 2010). Conversely, tasks in which the appraisal of emotional valence is evaluated (such as the valence judgment in the present study) are likely to have reduced reaction times (Calvo and Avero, 2009). Regarding accuracy findings, we observed that Picture task and Easy bar task were easier in comparison to the high load task, as expected. Subjects were slower and less accurate, indicating attention maintenance on this Hard bar task.

In conclusion, we highlight the importance of considering that other variables may facilitate the attentional control of emotional processing. Currently, the discrepancy between evidence about the automaticity of emotion processing and its dependence on attention can be explained, at least in part, by individual differences in attention control processes. Herein, subjects that scored higher in the positive affect trait exhibited lower amygdala reactivity to distracting unpleasant pictures relative to neutral. Thus, the current study suggests that positive affect modulates the effect of attention on unattended unpleasant pictures, therefore attenuating unpleasant emotional processing.

## Conflict of Interest Statement

The authors declare that the research was conducted in the absence of any commercial or financial relationships that could be construed as a potential conflict of interest.
